# The Effect of the NbVO_x_ Synthesis Protocol on the Extractive Catalytic Oxidative Desulfurization of Dibenzothiophene

**DOI:** 10.3390/molecules30030551

**Published:** 2025-01-25

**Authors:** Katarzyna Stawicka, Julia Gajewska, Maria Ziolek, Maciej Trejda

**Affiliations:** Department of Heterogeneous Catalysis, Faculty of Chemistry, Adam Mickiewicz University, Uniwersytetu Poznańskiego 8, 61-614 Poznań, Poland; katarzyna.stawicka@amu.edu.pl (K.S.); safrinbear@gmail.com (J.G.); ziolek@amu.edu.pl (M.Z.)

**Keywords:** oxidative desulfurization, niobium, vanadium, mixed oxides

## Abstract

NbVO_x_ mixed oxides were synthesized, characterized, and evaluated as catalysts for the extractive catalytic oxidative desulfurization (ECODS) of dibenzothiophene (DBT) using acetonitrile as a solvent. The mixed oxides were prepared using two different vanadium precursors: ammonium metavanadate and vanadium(IV)-oxy acetylacetonate. These precursors influenced the acidic/basic properties and the concentration of oxygen vacancies in the resulting catalysts. The texture and surface properties of the synthesized materials were analyzed using nitrogen adsorption/desorption, X-ray diffraction (XRD), X-ray photoelectron spectroscopy (XPS), and UV–visible spectroscopy (UV-vis). Their catalytic activity was evaluated through the dehydration and dehydrogenation of 2-propanol and the ECODS of DBT. The mixed oxides synthesized with an excess of ammonium metavanadate (Nb:V = 1:2) demonstrated superior catalytic activity in removing DBT from the oil phase, achieving approximately 90% removal within 90 min at 60 °C. This enhanced activity is attributed to its higher acidity, greater concentration of oxygen vacancies, and the presence of vanadium peroxo ligands on its surface.

## 1. Introduction

Metal oxides are widely used in heterogeneous catalysis, serving either as the active phase or as a support material. Their attractiveness in both academic and industrial research stems from their tunable acid-base and redox properties, which can be tailored through synthesis protocols. Among metal oxides, transition metal oxides stand out as particularly promising candidates for industrial catalytic applications. This is due to their low synthesis costs, ease of regeneration, and excellent thermal stability. Furthermore, metal oxides applied as catalysts often enable high selectivity for the desired product while achieving high yields. As a result, these materials have found diverse applications in catalysis. They are effective in a variety of catalytic processes, including oxidation, dehydration, dehydrogenation, isomerization, alkylation, condensation, cycloaddition, hydroxylation, and transesterification [1,2,3].

The synthesis of complex metal oxides, formed by combining two or more metallic ions in varying ratios, allows for the modification of the structural, textural, and surface properties of metal oxides. These materials can be synthesized using various methods, such as sol–gel, wet impregnation, mechanical mixing, co-precipitation, and microwave irradiation. The resulting oxides can be classified as mixed metal oxides (A_x_B_y_O_z_), hetero-metal-doped metal oxides (B:A_x_O_y_), or composite metal oxides consisting of a mixture of single oxide phases (A_w_O_x_/B_y_O_z_). Complex metal oxides exhibit distinct properties compared to single oxides, including differences in acidic, basic, and redox properties, as well as structural and textural parameters [4]. For example, TiO_2_-ZrO_2_ shows a higher number and strength of both basic and acidic sites compared to the individual oxides. Additionally, this material demonstrates a larger surface area and better thermal stability [5]. Another example is the WO_3_/TiO_2_ system, where the tungsten oxide phase increases the strength of Lewis acid sites and contributes to the formation of Brønsted acid sites [6]. The significant role of complex metal oxides in catalysis has also been demonstrated in studies such as that by [7], where the ZnO-MgO phase exhibited a higher density of stronger basic species than the single oxides, along with an increase in specific surface area. As highlighted by the cited literature, the clear advantages of complex metal oxides over single oxides make them increasingly attractive as potential heterogeneous catalysts.

In recent years, catalytic oxidative desulfurization combined with an extraction step (ECODS) has gained significant attention due to its mild reaction conditions and effective removal of aromatic sulfur compounds, which are difficult to eliminate by the industrially used hydrodesulfurization (HDS) process. The selection of an appropriate catalyst, oxidizing agent, extraction solvent, and operating conditions greatly influences desulfurization efficiency and the associated industrial cost. A suitable solvent applied in ECODS should exhibit several important characteristics, including good solubility for sulfones (the reaction products), the ability to be recovered from the reaction mixture, and low solubility for hydrocarbon mixtures to minimize fuel fraction loss. Numerous studies have utilized highly polar solvents such as acetonitrile, N,N-dimethylformamide (DMF), methanol, butyrolactone, and N,N-dimethylpyrrolidone for extraction purposes in ECODS. Among these, acetonitrile has proven to be a highly effective extraction solvent for feeds containing DBT due to its comparatively low boiling point of 82 °C, as opposed to the boiling points of sulfones, which range from 277 to 677 °C. Consequently, acetonitrile is easier to separate and reuse [8,9,10,11,12,13].

Among the catalysts used in oxidative desulfurization, metal oxides are particularly attractive due to their low cost and good catalytic activity [2,3]. The Ce-Mo-O oxide has shown high activity in oxidative desulfurization (ODS) when oxygen is used as the oxidizing agent. With this catalyst, the removal of dibenzothiophene (DBT) reaches 97% at 100 °C [14]. Another catalyst, the bimetallic mixed oxide Co-Mo-O, achieves 92.2% DBT removal at 120 °C within 1 h. The high activity of this catalyst is attributed to the synergistic interaction between Co and Mo species [15]. In the liquid-phase ODS process, the mixed oxide WO_3_/Fe_3_O_4_ achieves 92.3% DBT removal at 100 °C for 4.5 h in n-octane solution, using tert-butyl hydroperoxide (TBHP) as the oxidizing agent [16]. On the other hand, the Nb_2_O_5_/Al_2_O_3_ catalyst, when using TBHP, shows lower effectiveness, with only 43.4% DBT removal at 110 °C for 3 h [17]. The most recent studies focused on the application of mixed oxides on ODS show, when hydrogen peroxide (H_2_O_2_) is used as the oxidizing agent, the SnO_2_–MoO_3_ catalyst achieves a nearly 100% DBT removal at 60 °C within 30 min [18]. Another type of catalyst containing cobalt, specifically flower-like cobalt–molybdenum mixed-oxide microspheres (CoMo-FMs), is employed as a catalyst for the aerobic oxidative desulfurization (AODS) of fuel. This catalyst achieves 100% removal of DBT at 100 °C within 4 h, using air as the oxidizing agent [19]. Cobalt and molybdenum are also used as active components, along with iron species, to synthesize Co-Fe-Mo mixed metal oxides for testing in AODS. The optimal catalyst demonstrates high efficiency in DBT removal at 100 °C within 3 h [20]. Molybdenum species are also combined with iron to synthesize mixed oxides (Fe/Mo) loaded on an aluminum-pillared clay (Al-PILC) support. This material achieves a 94% yield of DBTO_2_ after 5 h of reaction in the presence of an oxidation mixture of H_2_O_2_ and HCOOH [21]. Another example of active catalysts in ODS are composite layered double hydroxide–metal oxides (Ni/Al-TiO_2_ and Ni/Al-ZnO), which demonstrate nearly 100% removal of DBT at 30 °C [22].

Based on the data provided above, the aim of this study was to synthesize various mixed metal oxides based on niobium and vanadium for use as catalysts in the extractive catalytic oxidative desulfurization (ECODS) of dibenzothiophene. This process utilizes acetonitrile as a solvent, which efficiently extracts the formed sulfone from the fuel due to its higher polarity compared to DBT. The mixed oxides were synthesized using different molar ratio of Nb:V (1:2 and 2:1) and vanadium sources: ammonium metavanadate and vanadium(IV)-oxy acetylacetonate. The applied different molar ratio of Nb:V and different precursors of vanadium species influenced the acidic/basic properties of the catalysts and the concentration of oxygen vacancies. To the best of our knowledge, there are no existing data in the literature regarding the effect of the synthesis protocol for mixed niobium–vanadium oxides on their surface and textural properties. It was hypothesized that oxides with a higher concentration of oxygen vacancies would be more effective in the ECODS process due to improved interaction with hydrogen peroxide. The influence of the morphology, surface properties, and acid/base characteristics of the mixed oxides on their activity in the ECODS process was examined in detail.

## 2. Results and Discussion

In this study, mixed NbVO_x_ oxides were synthesized using different vanadium sources—ammonium metavanadate and vanadium(IV)-oxy acetylacetonate and varying Nb to V molar ratios of 1:2 and 2:1. This approach enabled the investigation into how the synthesis protocol affects the texture, structure, surface properties, and catalytic activity of the mixed oxides.

### 2.1. Characterization of the Oxides

The surface areas of the oxides obtained are relatively small, as summarized in Table 1. For most samples, they range between 3 and 5 m^2^/g, with one exception: the NbVO_x_-CA21 sample. For this oxide, NH_4_VO_3_ was used as the vanadium precursor, and niobium was used in excess. Consequently, this sample exhibits a surface area approximately five times larger than that of the other three samples.

**Figure 1:**
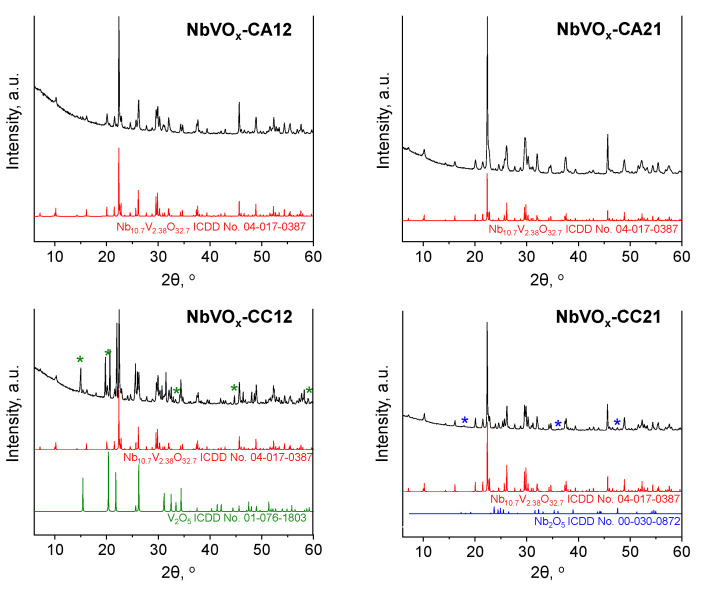
XRD patterns confirming the crystalline structure of NbVO_x_ mixed oxides (*—V_2_O_5_ crystalline phase, *—Nb_2_O_5_ crystalline phase).

The wide-angle XRD patterns of the synthesized catalysts, shown in Figure 1, confirm the successful formation of crystalline mixed oxides. The patterns for NbVO_x_-CA12 and NbVO_x_-CA21, synthesized from NbCl_5_ and NH_4_VO_3_ precursors, exhibit reflections corresponding to the Nb_10.7_V_2.38_O_32.7_ crystalline phase (ICDD No. 04-017-0387). Notably, no additional reflections associated with crystalline Nb_2_O_5_ or V_2_O_5_ phases are observed in these materials. In contrast, for samples synthesized using OV(acac)_2_ as the vanadium precursor, reflections corresponding to crystalline niobium(V) oxide (ICDD No. 00-030-0872) and vanadium(V) oxide (ICDD No. 01-076-1803) are detected. For these samples, the single oxides Nb_2_O_5_ and V_2_O_5_ form when NbCl_5_ or OV(acac)_2_ are used in excess during synthesis, respectively. These XRD results suggest that a higher purity NbVO_x_ phase can be achieved when niobium species are present in the cation position and vanadium species in the anion position of the precursors used for synthesis. This phenomenon appears to be independent of the Nb:V molar ratio.

**Figure 2:**
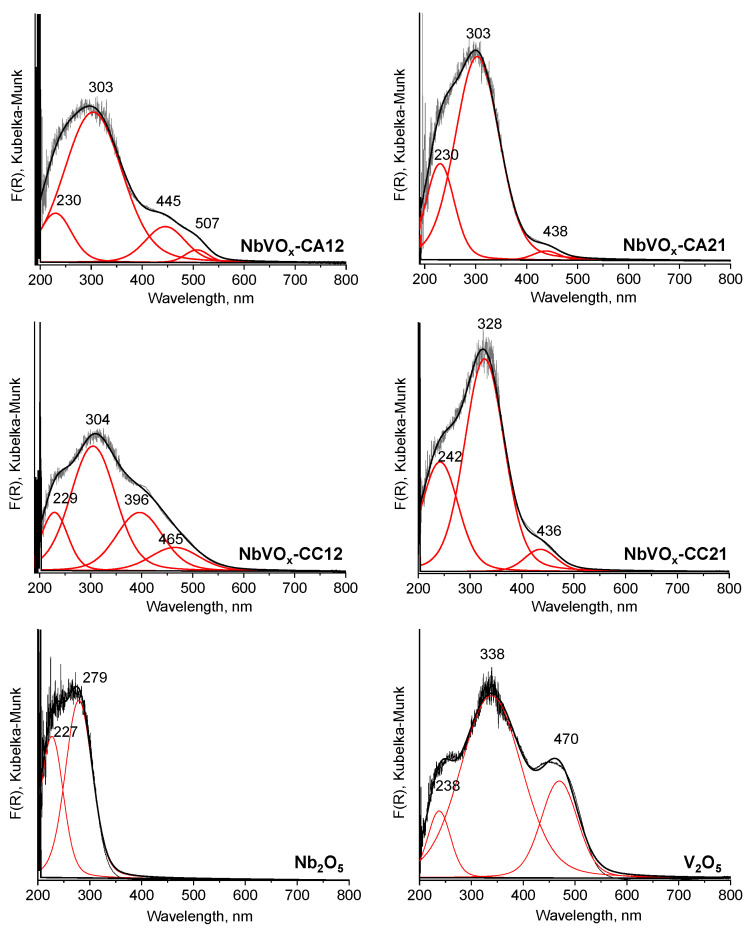
UV-vis spectra of NbVO_x_ mixed oxides, Nb_2_O_5_ and V_2_O_5_.

The UV-vis spectra in Figure 2 confirm the successful synthesis of the mixed NbVO_x_ oxides, as they differ significantly from the spectra of individual oxides Nb_2_O_5_ and V_2_O_5_.

The UV-vis spectra of the NbVO_x_ samples exhibit bands at approximately 229–242 nm, 303–328 nm, and 438–445 nm. The first two bands are associated with tetrahedrally- and octahedrally coordinated niobium species, respectively [23]. These bands may overlap with those attributed to isolated V⁵⁺ tetrahedrally coordinated species and monomeric tetrahedral vanadium species, respectively [24]. The band at 438–445 nm is likely due to charge transfer between oxygen and vanadium in octahedral coordination [25]. Interestingly, these bands (except for the one at approximately 436 nm) are red-shifted by approximately 13–24 nm in the spectrum of NbVO_x_-CC21, suggesting a different local environment for these species compared to those in the other NbVO_x_ samples. Notably, the UV-vis spectra of NbVO_x_-CA12 and NbVO_x_-CC12 reveal additional bands. The spectrum of NbVO_x_-CA12 shows a band at 507 nm, while NbVO_x_-CC12 exhibits bands at 396 nm and 465 nm. According to the literature, the 507 nm band is characteristic of the charge transfer in peroxo ligands [VO(O–O)_2_]^−^ [26], the 396 nm band is attributed to vanadium aggregates in octahedral coordination, and the 465 nm band is associated with bulk vanadium oxide. It is particularly noteworthy that the band attributed to vanadium(V) oxide was only detected in the UV-vis spectrum of NbVO_x_-CC12, which correlates with the XRD data, where typical V_2_O_5_ reflections are observed only for this sample [26].

The oxidation states of the metals and their concentrations in the synthesized mixed oxides were determined by XPS measurements, as shown in Table 1 and Figure S1. Niobium in the NbVO_x_ samples is present in the +V oxidation state, while the vanadium species exhibit two oxidation states, +V and +IV [27,28,29]. The data in Table 1 show that mixed oxides synthesized with a 2:1 Nb:V molar ratio have a higher concentration of vanadium species in the lower oxidation state. This is accompanied by a shift in the binding energies (BE) of niobium species to higher values. The shift in the BE of niobium species is also accompanied by a shift in vanadium species to higher BE values. The type of vanadium precursor used in the synthesis of the mixed oxides also influences the BE of niobium and vanadium. When OV(acac)_2_ is used as the source of vanadium species, the BE of niobium and vanadium in NbVO_x_ samples shifts to higher values. These results clearly demonstrate the impact of both the Nb:V molar ratio (1:2 or 2:1) and the type of vanadium precursor (with vanadium species in the cation or anion position) on the electronic state of the metals in the mixed oxides.

The XP spectra of the O1s region provide valuable information regarding the concentration of oxygen vacancies in the synthesized mixed oxides (Figure S1 and Table 1). Three types of oxygen species can be distinguished in the spectrum. The dominant species is characteristic of lattice oxygen in the oxide appearing at lower binding energy. The band appearing at higher binding energies, ca. 531 eV, corresponds to less electron-rich oxygen species [30], for instance oxygen associated with vacancies [29]. The less intensive band at ca. 533 eV, according to the literature, can be associated with the hydroxyl species [31]. It is evident that the samples containing lower amounts of niobium exhibit a higher concentration of oxygen vacancies, with values ranging from 11.1% to 20.4%. Both the Nb:V molar ratio and the type of vanadium precursor used in the synthesis influence the formation of oxygen vacancies. When the Nb:V molar ratio is 1:2, a higher concentration of oxygen vacancies is typically formed. The type of vanadium precursor also affects the formation of oxygen vacancies. A higher concentration of oxygen vacancies is observed when NH_4_VO_3_ is used as the vanadium source. The highest concentration of oxygen vacancies, 20.4%, is found in NbVO_x_-CA12. Notably, only in the UV-vis spectrum of this material is a band assigned to peroxo ligands observed.

The acid/base properties of the NbVO_x_ samples were investigated using the dehydration and dehydrogenation of 2-propanol as a test reaction. The dehydration of the alcohol at acidic sites leads to the formation of propene and diisopropyl ether, while dehydrogenation, which occurs at basic sites, results in acetone. The cooperation between acid and basic sites also leads to the formation of diisopropyl ether [32]. The initial information regarding the acid/base properties of the obtained oxides can be inferred from their catalytic activity, specifically the conversion of 2-propanol. The results presented in Figure S2 clearly demonstrate differences between samples prepared with various vanadium precursors. In general, when NH_4_VO_3_ is used for oxide preparation, higher conversion rates are observed, with the highest activity exhibited by the material prepared with an excess of niobium, i.e., NbVO_x_-CA21. Notably, this catalyst also demonstrates superior activity at lower reaction temperatures.

**Figure 3:**
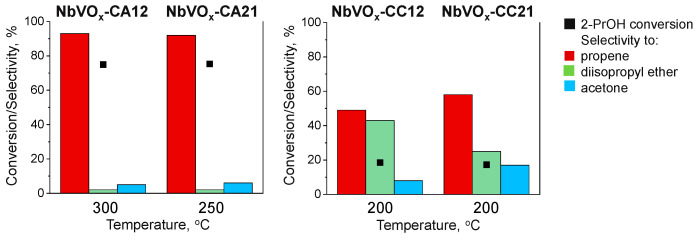
The results of 2-propanol dehydration and dehydrogenation (■) 2-propanol conversion, selectivity to propene (■), diisopropyl ether (■), and acetone (■).

To gain deeper insight into the nature of the active sites, the selectivity toward reaction products should be analyzed for materials with similar 2-propanol conversion rates. Figure 3 provides such a comparison. For the catalysts with the highest activity, namely NbVO_x_-CA21 and NbVO_x_-CA12, no significant differences in selectivity are observed. Thus, it can be concluded that these catalysts likely possess similar types of acid centers, and the difference in activity is more likely attributed to the number of active sites. However, some differences are noted when OV(acac)_2_ is used as the vanadium precursor in combination with an excess of niobium (Nb:V = 1:2). For these catalysts, slightly higher selectivity toward propene and acetone is observed for NbVO_x_-CC21.

### 2.2. Extractive Catalytic Oxidative Desulfurization of Dibenzothiophene

The catalytic activity of the NbVO_x_ samples in extractive catalytic oxidative desulfurization (ECODS) was assessed in the liquid phase using a mixture of dibenzothiophene in dodecane (DBT, 500 ppm), hydrogen peroxide as the oxidizing agent, and acetonitrile as the solvent. The DBT concentration in the dodecane solution was monitored by gas chromatography (GC) throughout the reaction.

**Figure 4:**
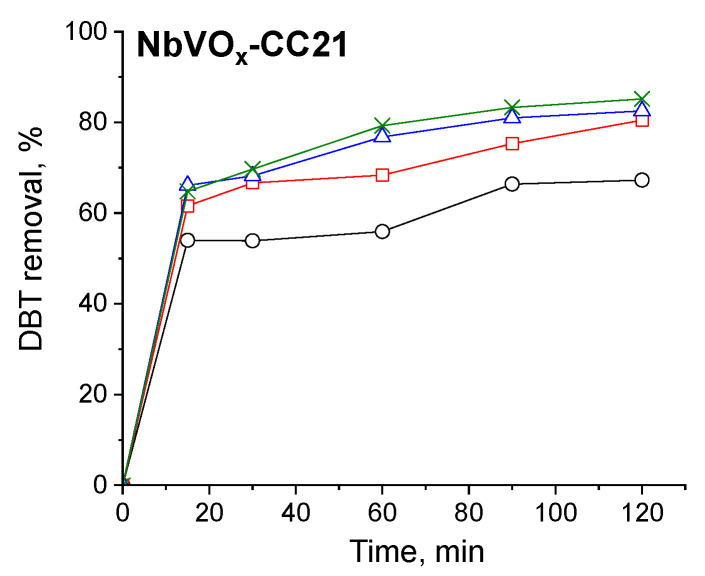
The impact of the mass of catalyst on DBT removal: (○) 0.031 wt.%; (□) 0.062 wt.%; (∆) 0.125 wt.%; (×) 0.188 wt.%.

First, to determine the appropriate catalyst mass for the reaction, different amounts (0.031–0.188 wt.%) of selected materials were tested. The results, shown in Figure 4, reveal that increasing the catalyst amount leads to a higher DBT removal. The most significant increase in activity occurs when the catalyst mass is raised from 0.031 to 0.062 wt.%. Further increases in catalyst mass result in only a marginal improvement in DBT removal, particularly for the NbVO_x_-CC21 sample. Therefore, a catalyst mass of 0.125 wt.% was chosen for the subsequent reaction tests.

**Figure 5:**
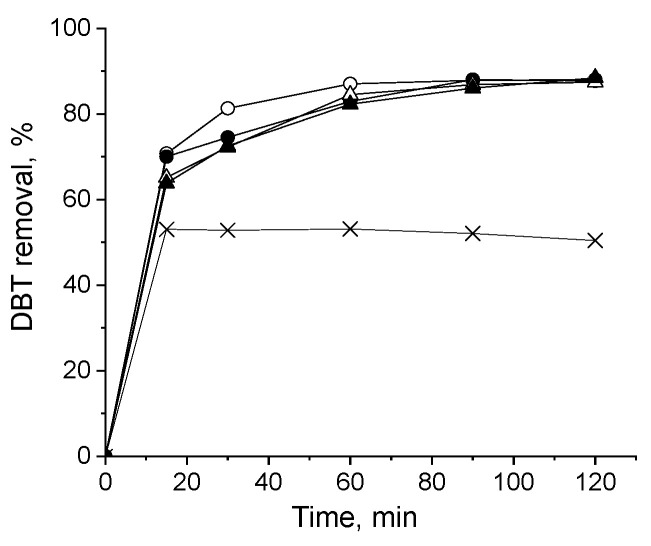
The catalytic activity of the materials obtained in extractive oxidative desulfurization: (×) without catalyst, (○) NbVO_x_-CA12, (●) NbVO_x_-CC12, (∆) NbVO_x_-CA21, (▲) NbVO_x_-CC21. Oxidation conditions: H_2_O_2_:S = 6:1, wt.%(cat.) = 0.125, 60 °C, 1000 rpm, 120 min.

**Figure 6:**
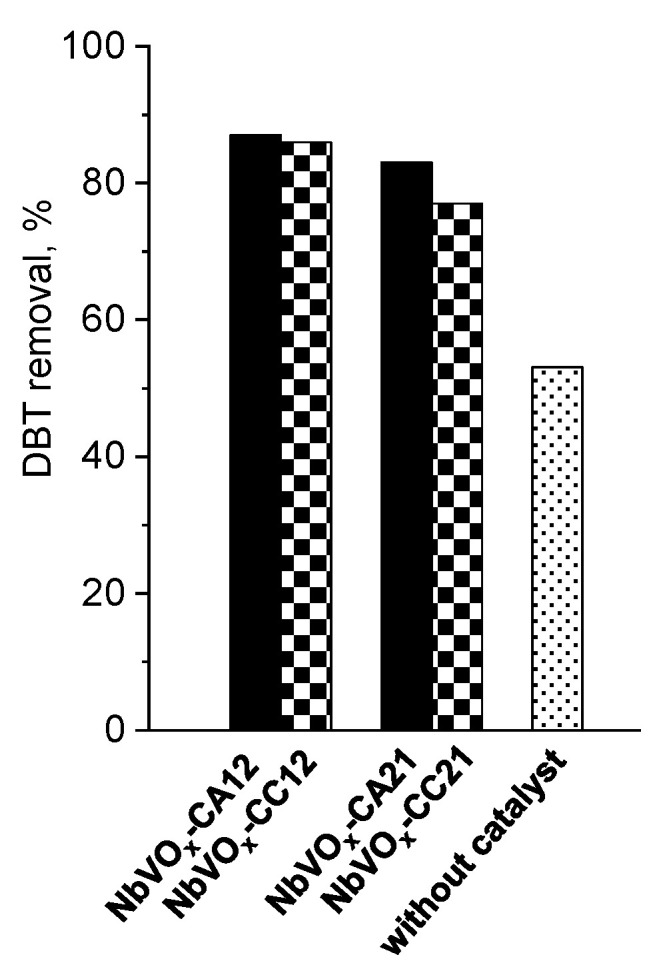
The catalytic activity of the materials obtained in extractive oxidative desulfurization. Oxidation conditions: H_2_O_2_:S = 6:1, wt.%(cat.) = 0.125, 60 °C, 1000 rpm, 60 min.

Figure 5 presents the results of the ECODS process using all synthesized catalysts with an H_2_O_2_/DBT molar ratio of 6 depending on reaction time, whereas Figure 6 summarizes the DBT removal within 1 h. The reactions were conducted with 0.125 wt.% of catalyst for 120 min. Taking into account the boiling point of acetonitrile and the previous results reported in [33], the process was performed at 60 °C. The use of acetonitrile as the extractive solvent resulted in a decrease in the concentration of dissolved dibenzothiophene in dodecane by approximately 50%, with the reaction time having little effect on this process. The addition of 0.125 wt.% mixed oxides significantly increased DBT removal, achieving up to 80–90% removal after approximately 90 min. Prolonging the reaction time further did not lead to additional increases in DBT removal. Notably, sulfone, the main reaction product, was only detected in the acetonitrile solution. Of the tested catalysts, those synthesized using NH_4_VO_3_ as the vanadium source exhibited a higher activity than those prepared with the use of vanadium(IV)-oxy acetylacetonate. It is well established that the ODS process is more efficient with catalysts that possess both acidic and redox sites [33,34,35]. Therefore, the superior activity of NbVO_x_-CA12 can likely also be attributed to their higher acidity, as confirmed by the 2-propanol dehydration and dehydrogenation tests, as well as their higher concentration of oxygen vacancies, as shown by XPS measurements. The most active catalyst among the synthesized oxides, especially at the beginning of the process, was NbVO_x_-CA12, which was prepared from NbCl_5_ and NH_4_VO_3_ sources. This sample is the only one that contains peroxo ligands [VO(O-O)_2_]^−^, as identified by UV-vis spectroscopy. This suggests that the presence of these species on the surface of the mixed oxides plays an important role in the efficient removal of DBT.

**Figure 7:**
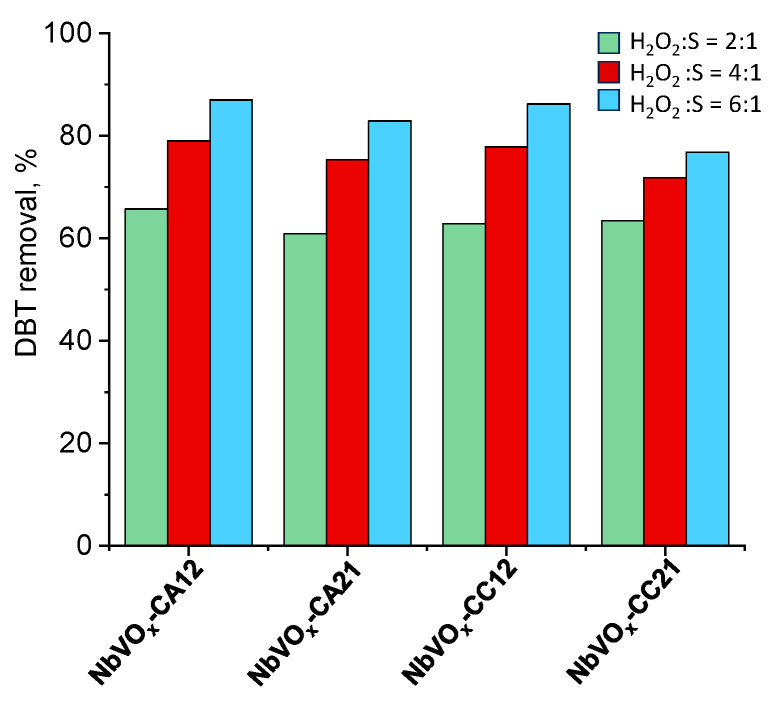
Influence of hydrogen peroxide amount on DBT conversion. Oxidation conditions: wt.%(cat.) = 0.125, 60 °C, 1000 rpm, 60 min.

Not surprisingly, decreasing the molar ratio of hydrogen peroxide to DBT in the reaction mixture from 6 to 4 and 2 results in a reduction in DBT removal by approximately 20% (Figure 7 and Figure S3). This trend can be observed for all tested catalysts. This decrease is likely due to an insufficient amount of hydrogen peroxide in the reaction medium, which limits the formation of the required amount of reactive oxygen species (ROS) through the interaction of H_2_O_2_ with the mixed oxides. It is well-established that niobium can generate various ROS, such as peroxo, superoxo, and hydroxyl radicals, through their interaction with hydrogen peroxide [36,37,38,39,40]. A higher concentration of ROS leads to more efficient oxidation of the substrate, such as DBT. However, with lower concentrations of H_2_O_2_, fewer ROS are generated, resulting in less efficient oxidation of DBT to sulfoxide, which is not effectively removed from the dodecane solution.

**Figure 8:**
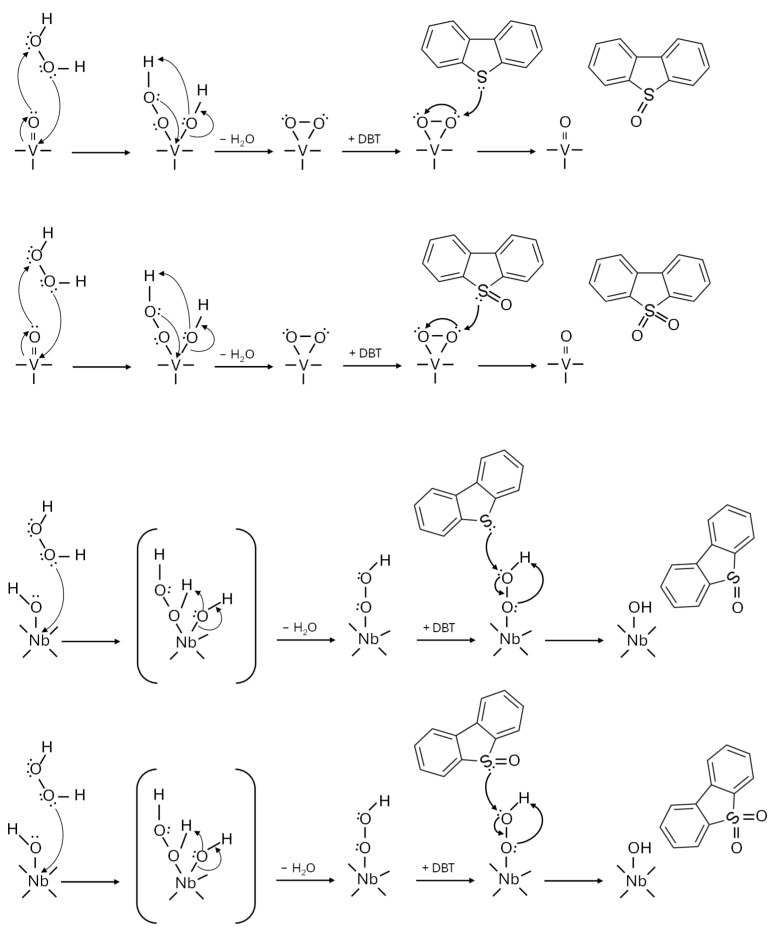
Proposed mechanism of DBT oxidation on Nb and V active sites.

For one of the selected materials, NbVOx-CC21, additional reactions were performed in the presence of radical scavengers to investigate the mechanism further. In this study, 2-propanol was used to target hydroxyl radicals, while benzoquinone was employed to scavenge superoxide anion radicals. The results indicated that the addition of radical scavengers did not influence the efficiency of DBT removal. This suggests that the aforementioned radicals are not involved in the DBT oxidation process. Instead, it can be inferred that peroxy species generated from H_2_O_2_ are primarily responsible for the oxidation reaction, which is consistent with previous literature [41,42]. The proposed formation of peroxy species on both Nb and V active sites is illustrated in Figure 8.

Table 2 compares the catalytic activity in ECODS using hydrogen peroxide as the oxidizing agent and acetonitrile as the solvent. The comparison includes NbVO_x_-CA12 from this study and mixed metal oxides reported in the literature. It is evident that NbVO_x_-CA12 demonstrates notable efficiency in removing DBT from the oil phase, comparable to other listed catalysts.

## 3. Materials and Methods

### 3.1. Materials

Niobium(V) chloride (>99%), vanadium(IV)-oxy acetylacetonate (>98%), ammonium metavanadate (>99%), dibenzothiophene (98%), and dodecane and hydrogen peroxide (30%) were purchased from Sigma-Aldrich (St. Louis, MO, USA). 2-propanol (>99%) was purchased from Chempur (Poland). Ammonium hydroxide (25%) was purchased from Stanlab (Poland). Methanol (>99%) was purchased from EUROCHEM BGD (Poland).

### 3.2. Preparation of Catalysts

#### 3.2.1. Synthesis of NbVO_x_-CA12 and NbVO_x_-CA21

**Figure 9:**
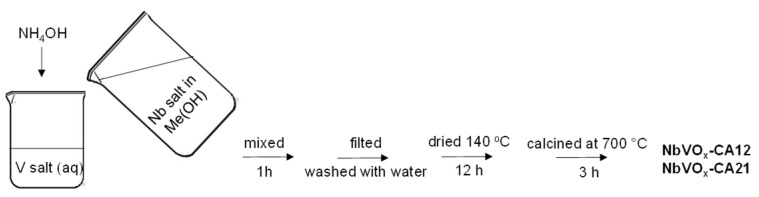
The scheme of synthesis protocol of NbVO_x_-CA12 and NbVO_x_-CA21.

Initially, 2.0 g of ammonium metavanadate was dissolved in 600 mL of distilled water and mixed for 24 h (Figure 9). Next, 2.31 g of niobium(V) chloride was dissolved in 20 mL of anhydrous methanol. The molar ratio of Nb:V was assumed to be 1:2. For the synthesis of the NbVO_x_ oxide with a Nb:V molar ratio of 2:1, 1.0 g of ammonium metavanadate and 4.62 g of niobium(V) chloride were used. In the next step, the prepared NbCl_5_ solution was poured into the ammonium metavanadate solution, maintaining the pH at 7 by adding aqueous ammonia. The pH of the synthesis mixture was kept at 7 throughout the addition of the niobium precursor solution by continued addition of aqueous ammonia. The final mixture was stirred for 1 h. The resulting precipitate was then filtered, washed with 300 mL of distilled water, and dried at 140 °C for 12 h. Finally, the material was calcined at 700 °C for 3 h with a temperature ramp of 5 °C/min. The samples obtained using this method were labeled as NbVO_x_-CA12 and NbVO_x_-CA21, depending on the metal precursor used (niobium in the cation (C) of the NbCl_5_ and vanadium in the anion (A) of the NH_4_VO_3_) and the Nb:V molar ratio (1:2—12 and 2:1—21).

#### 3.2.2. Preparation of NbVO_x_-CC12 and NbVO_x_-CC21

**Figure 10:**
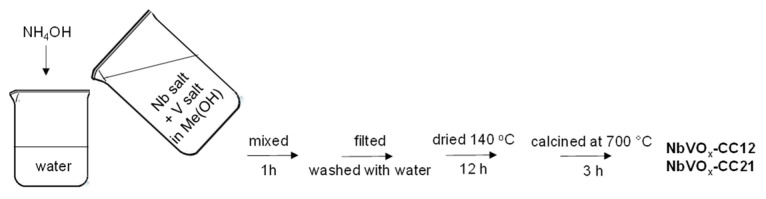
The synthesis protocol of NbVO_x_-CC12 and NbVO_x_-CC21.

Initially, 4.074 g of niobium(V) chloride and 8.0 g of vanadium(IV)-oxy acetylacetonate were dissolved in 40 mL of anhydrous methanol, with the assumed molar ratio of Nb:V set at 1:2 (Figure 10). For the synthesis of NbVO_x_ oxide with a Nb:V molar ratio of 2:1, 4.0 g of vanadium(IV)-oxy acetylacetonate and 8.148 g of niobium(V) chloride were used. Next, the prepared solution of metal precursors was poured into 100 mL of distilled water, and the pH was first adjusted to 7 by adding aqueous ammonia. The pH of the synthesis mixture was maintained at 7 throughout the addition of the precursors solution by continuing to add aqueous ammonia. The final mixture was stirred for 1 h. The resulting precipitate was filtered, washed with 600 mL of distilled water, and dried at 140 °C for 12 h. Finally, the material was calcined at 700 °C for 3 h with a temperature ramp of 5 °C/min. The samples obtained through this method were labeled as NbVO_x_-CC12 and NbVO_x_-CC21, depending on the metal precursor used (niobium in the cation (C) of the NbCl_5_ and vanadium in the cation (C) of the OV(acac)_2_) and the molar ratio (1:2—12 and 2:1—21).

### 3.3. Catalyst Characterization

The obtained catalysts were characterized using low-temperature nitrogen adsorption/desorption, XRD, UV-vis, and XPS measurements.

The N_2_ adsorption/desorption isotherms were measured using an ASAP 2020 apparatus with a liquid nitrogen temperature of −196 °C and in a relative pressure range of p/p_0_ 0.01 to 0.99. Prior to the measurements, the ca. 0.4 g of catalysts were outgassed under vacuum at 300 °C for 8 h. The surface area was determined using the Brunauer–Emmett–Teller (BET) method, while the pore volume and pore diameter were estimated using the Barrett–Joyner–Halenda (BJH) method.

XRD patterns were obtained on a Bruker AXS D8 Advance diffractometer using Cu K_λ_ radiation (λ = 0.154 nm), with a step of 0.05° in the range of 2θ = 6–60°.

UV-vis spectra in the range of 800–190 nm were recorded using a Varian–Cary 300 Scan UV-vis spectrophotometer, with SPECTRALON used as the reference sample. Prior to the measurements, the samples were dried overnight at 100 °C.

XPS measurements were performed using an ultra-high vacuum (UHV) system (Specs) equipped with a monochromatic microfocused Al K_λ_ X-ray source (1486.6 eV). Binding energies were referenced to the C 1s peak at 284.6 eV.

### 3.4. Test Reactions

The acid/base surface properties of the mixed oxides were examined through 2-propanol dehydration and dehydrogenation, while their catalytic activity was evaluated in the extractive catalytic oxidative desulfurization (ECODS) of dibenzothiophene (DBT) using acetonitrile as the solvent.

The dehydration and dehydrogenation of 2-propanol (2-PrOH) were conducted using a microcatalytic pulse reactor (Ø 6 mm, length 80 mm) connected to the catalyst inlet and the column of an SRI 310 chromatograph. A 0.1 g portion of granulated oxide (0.5 < Ø < 1.0 mm) was activated at 350 °C (heating rate: 10 °C/min) for 2 h under a nitrogen flow of 40 mL/min. The conversion of 2-PrOH was studied at 150–300 °C using 3 μL pulses of 2-PrOH, with nitrogen (65 mL/min) as the carrier gas. The substrate was vaporized before passing through the catalyst bed. The reaction mixture was separated using a 2 m column packed with Carbowax 400 on Chromosorb W (80–100 mesh) at 65 °C under a nitrogen flow of 40 mL/min and was detected by a flame ionization detector (FID).

The extractive catalytic oxidative desulfurization (ECODS) of dibenzothiophene (DBT) was performed in a glass reactor using an EasyMax Workstation. The ECODS experiments were conducted using the following mixture: 500 ppm of DBT in dodecane (5 mL), acetonitrile (5 mL), 0.125 wt.% of catalyst, and H_2_O_2_ (30%; H_2_O_2_/S ratios of 6, 4, and 2). Prior to the reaction, the oxides were preheated in an oven at 400 °C for 8 h to remove moisture from their pores. The standard ECODS process was carried out for 120 min at 60 °C. The effects of reaction time, the amount of hydrogen peroxide, and the catalyst mass on DBT removal were analyzed. The DBT removal was calculated using the following equation:

(1) $$\mathrm{Conversion}\;\mathrm{of}\;\mathrm{DBT}=\frac{{(C}_{0}-\;C)}{C_{0}}\times \;100\%$$

where C_0_ means the concentration of DBT at the beginning of the reaction, C means the concentration of DBT during the reaction.

A gas chromatograph (Trace 1300, Thermo Scientific) equipped with a 30 m DB-1 column and a flame ionization detector (FID) was used to monitor DBT concentration during the ECODS process. Helium was used as the carrier gas. The injector and detector temperatures were set to 250 °C and 280 °C, respectively. The column temperature was initially set at 80 °C for 3 min, followed by an increase to 300 °C at a ramp rate of 10 °C/min.

## 4. Conclusions

NbVO_x_ oxides were synthesized using different Nb:V molar ratios (1:2 or 2:1) and two vanadium precursors, ammonium metavanadate (NH_4_VO_3_) and vanadium(IV)-oxy acetylacetonate (OV(acac)_2_), which provide vanadium in either the anionic or cationic position, respectively. The resulting catalysts were characterized and applied in the extractive catalytic oxidative desulfurization (ECODS) of dibenzothiophene (DBT) in a three-phase system, with acetonitrile as the solvent. Using NH_4_VO_3_ as the vanadium source resulted in the mixed NbVO_x_ oxides, without contamination from Nb_2_O_5_ or V_2_O_5_ phases. The highest concentration of V^4+^ species was observed in samples synthesized with a Nb:V molar ratio of 2:1, whereas a higher concentration of oxygen vacancies was seen in the 1:2 molar ratio. The NbVO_x_-CA12 sample exhibited the highest concentration of oxygen vacancies. It was also the only sample to show peroxo ligands on its surface. The NbVO_x_ materials obtained using NH_4_VO_3_ demonstrated higher acidity, which was reflected in their superior activity in the dehydration and dehydrogenation of 2-propanol. These samples allowed us to obtain sufficient activity in the extractive oxidative desulfurization of dibenzothiophene with up to 90% of DBT removal after 90 min, using only 0.125 wt.% of the catalyst, particularly for NbVO_x_-CA12. The enhanced activity of these samples is likely due to the higher concentration of oxygen vacancies (with peroxo ligands detected in NbVO_x_-CA12) and their higher acidity as well as higher concentrations of vanadium at the +4 oxidation state.

## Figures and Tables

**Table 1:** BET surface area and the composition of mixed oxides determined by XPS measurement.

	Specific Surface Area [m^2^g^−1^]			XPS				
Catalyst		Nb		V		O		
		3d 5/2 (Nb^5+^)	3d 3/2 (Nb^5+)^	2p 3/2 (V^5+^)	2p 3/2 (V^4+^)	1s (OH) *	1s (OV) *	1s (LA) *
NbVO_x_-CA12	3.4	209.5	206.7	517.1 (69.3%)	516.5 (30.7%)	533.0 (7.9%)	531.2 (20.4%)	529.8 (71.7%)
NbVO_x_-CA21	14.7	209.6	206.8	517.3 (65.5%)	516.5 (34.5%)	533.0 (3.7%)	530.9 (19.7%)	529.9 (76.6%)
NbVO_x_-CC12	3.0	209.7	207.0	517.4 (60.1%)	517.0 (39.9%)	532.8 (7.5%)	531.0 (14.8%)	530.0 (77.7%)
NbVO_x_-CC21	4.8	209.8	207.0	517.5 (41.9%)	517.1 (58.1%)	532.5 (4.0%)	531.3 (11.1%)	530.1 (84.9%)

*—OH—hydroxyl species; OV—oxygen associated with vacancies; LA—lattice oxygen.

**Table 2:** The comparison of activity in ECODS of mixed metal oxides.

Catalyst	Catalyst Mass, mg	Solvent	H_2_O_2_/S	Time, min.	Temp., °C	S Removal, %	Ref.
Mo-TiO_2_-SO_3_H	37.5	MeCN	2	10	80	100	[43]
Mo12bh (MoO_x_/Al_2_O_3_)	100	MeCN	6	30	60	97	[44]
W17 (W/γ-Al_2_O_3_)	100	MeCN	6	30	60	98	[45]
20wt%WO_x_/meso-SnO_2_	100	MeCN	5	60	50	100	[46]
NbVO_x_-CA12	4.7	MeCN	6	90	60	90	This work

## Data Availability

The raw data supporting the conclusions of this article will be made available by the authors on request.

## Supplementary Materials

The following supporting information can be downloaded at: https://www.mdpi.com/article/10.3390/molecules30030551/s1. Figure S1: XP spectra of NbVO_x_ oxides; Figure S2: The results of 2-propanol dehydration and dehydrogenation (■); 2-propanol conversion and selectivity to propene (■); and diisopropyl ether (■) and acetone (■). Figure S3: Influence of hydrogen peroxide amount and reaction time on DBT removal: H_2_O_2_:S = 2:1 (○), 4:1 (□), 6:1 (∆). Oxidation condition: wt.%(cat.) = 0.125, 60 °C, 1000 rpm, 120 min.
