# Supplementary material for: The Effect of the NbVOx Synthesis Protocol on the Extractive Catalytic Oxidative Desulfurization of Dibenzothiophene
Source: Molecules. 2025 Jan 25;30(3):551. doi: 10.3390/molecules30030551 (PMC11820594; doi:10.3390/molecules30030551)
Supplement: Supplementary file 1 [file molecules-30-00551-s001.zip › molecules-3405946-supplementary.pdf]

The Effect of the $NbVO_x$ Synthesis Protocol on the Extractive Catalytic Oxidative Desulfurization of Dibenzothiophene

Katarzyna Stawicka, Julia Gajewska, Maria Ziolek, Maciej Trejda*

Adam Mickiewicz University, Poznań, Faculty of Chemistry, Department of Heterogeneous Catalysis, Uniwersytetu Poznańskiego 8, 61-614 Poznań, Poland

*Correspondence: tmaciej@amu.edu.pl; Tel.: 0048 61 8291686

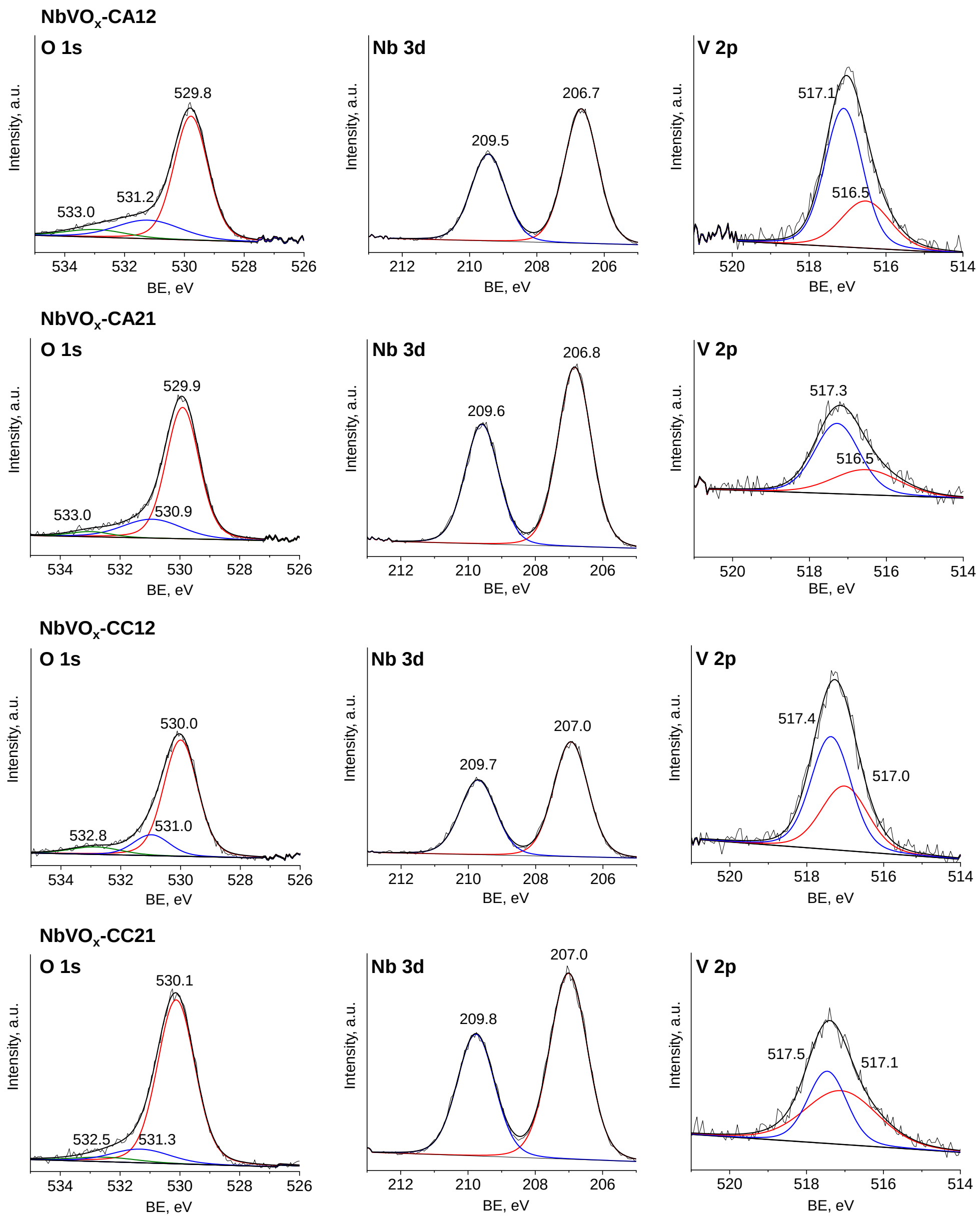

Figure S1. XP spectra of $NbVO_x$ oxides.

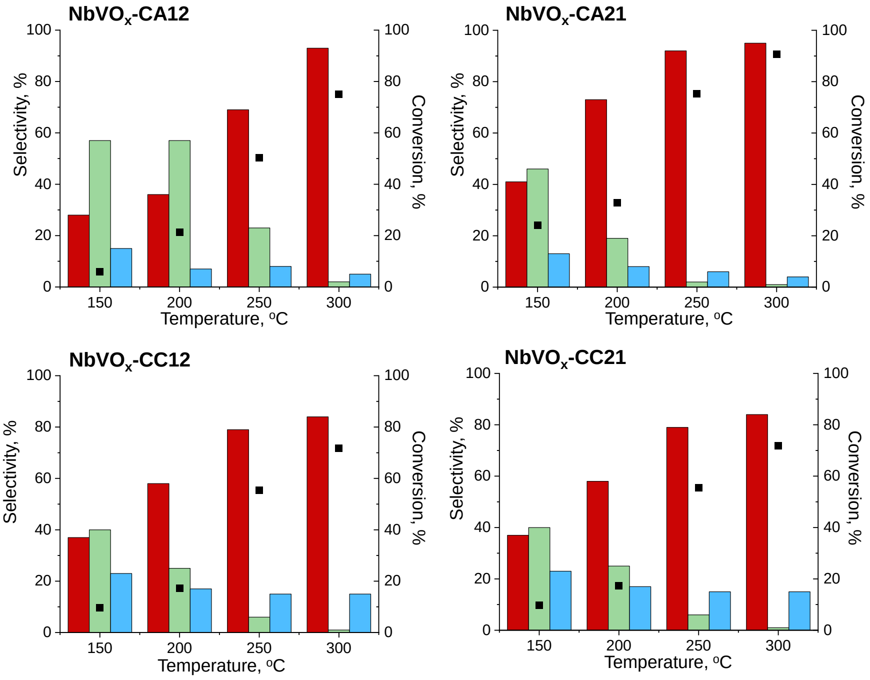

Figure S2. The results of 2-propanol dehydration and dehydrogenation (■) 2-propanol conversion, selectivity to propene (■), diisopropyl ether (■) and acetone (■).

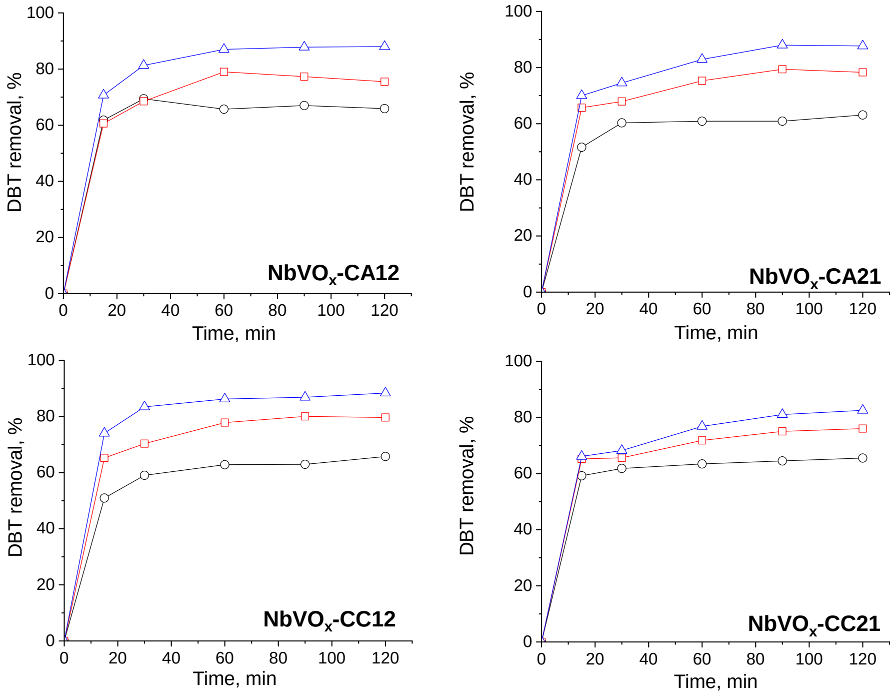

Figure S3. Influence of hydrogen peroxide amount and reaction time on DBT removal: H_2O_2:S = 2:1 (○), 4:1(□), 6:1 (Δ). Oxidation condition: wt.%(cat.) = 0.125, 60°C,1000 rpm, 120 min.
